# Functional imaging: what evidence is there for its utility in clinical trials of targeted therapies?

**DOI:** 10.1038/bjc.2011.579

**Published:** 2012-01-26

**Authors:** N Tunariu, S B Kaye, N M deSouza

**Affiliations:** 1 Section of Clinical Magnetic Resonance, The Institute of Cancer Research and Royal Marsden NHS Foundation Trust, Sutton, Surrey SM2 5PT, UK; 2 Drug Development Unit, The Institute of Cancer Research and Royal Marsden NHS Foundation Trust, Sutton, Surrey SM2 5PT, UK

**Keywords:** phase I trial, imaging biomarker, targeted agents, multiparametric imaging

## Abstract

Key issues in early clinical trials of targeted agents include the determination of target inhibition, rational patient selection based on pre-treatment tumour characteristics, and assessment of tumour response in the absence of actual shrinkage. There is accumulating evidence that functional imaging using advanced techniques such as dynamic contrast enhanced (DCE)-magnetic resonance imaging (MRI), DCE-computerised tomography (CT) and DCE-ultrasound, diffusion weighted-MRI, magnetic resonance spectroscopy and positron emission tomography-CT using various labelled radioactive tracers has the potential to address all three. This article reviews this evidence with examples from trials using targeted agents with established clinical efficacy and summarises the clinical utility of the various techniques. We therefore recommend that input from specialist radiologists is sought at the early stages of trial design, in order to ensure that functional imaging is incorporated appropriately for the agent under study. There is an urgent need to strengthen the evidence base for these techniques as they evolve, and to ensure standardisation of the methodology.

An increasing knowledge about the major pathways dysregulated in cancer has resulted in a large array of targeted pathway inhibitors submitted for phase I trials. Recent figures suggest that <10% of new molecules reach the market primarily because of a lack of demonstrable clinical activity (Kola and Landis, 2004). Other contributing factors include lack of appropriate quantitative imaging methods to guide both preclinical and clinical development. The standard imaging assessment of tumour response relies on size measurements, which, with predominantly cytostatic targeted agents, may not reflect the drug effect. The wide therapeutic index of targeted agents, non-linear relationship between dose and effect and non-mechanism related toxicity may require definition of an optimal biological dose rather than a maximal tolerated dose (MTD). Also, many of these agents may prove to be more effective in combination therapy either with synergistic targeted agents or with chemotherapy and the minimum biologically active dose needs also to be determined. Moreover, because targeted agents are approved for cancer treatment largely based on marginal benefits shown in clinical trials, it means that a large percentage of patients may merely suffer toxicity without therapeutic benefit.

Functional imaging biomarkers have the potential to quantify biological characteristics of tumours and measure on-target and off-target effects that indicate early likelihood of response to a specific therapy, which can then be used to guide the optimal biological dose and drug schedule. Serial, non-invasive assessments of whole tumour are possible. This is particularly important in the context of inter and intra-patient tumour heterogeneity as different parts of the tumour and primary *vs* metastatic lesions may be biologically different and these characteristics may change with treatment. This article reviews currently available functional imaging methods and discusses their potential for stratifying patients for targeted therapies and assessment of tumour response by measuring on-target and off-target effects. PubMed searches for the terms phase I trial, RECIST, antiangiogenic, cytostatic, molecular imaging, dynamic contrast enhanced (DCE)-magnetic resonance imaging (MRI), diffusion weighted (DW)-MRI, DCE-ultrasound (US), FDG-positron emission tomography (PET), treatment response and derivative references, were performed. Abstracts and articles judged most relevant to the goals of this report were reviewed with emphasis on limitations and strengths of the imaging approaches to treatment response assessment.

## Functional imaging measurements and terminology

**Figure 1:**
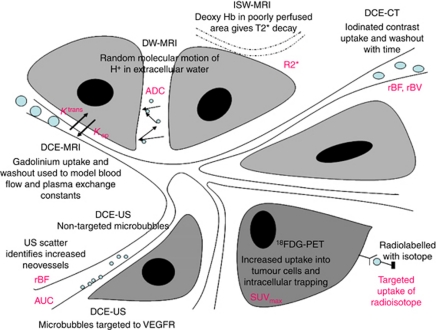
Schematic showing the currently most used functional techniques in the clinic and illustrating their mechanism of action. The output measures from each of these are shown in pink. Dynamic contrast enhanced MRI with output measured as rate constants (*K*^trans^ and *K*_ep_) of gadolinium transfer between intravascular and extravascular compartments measured in ml min^–1^. Diffusion weighted MRI with measured apparent diffusion coefficient (ADC) measured in mm^2^ s^–1^. Intrinsic susceptibility weighted MRI with output the relaxation rate constant R2^*^ measured in s^−1^. Dynamic contrast enhanced CT, with output of relative blood flow (rBF) and relative blood volume (rBV) measured in ml min^–1^ and ml ,respectively. Parameters in PET are measured as maximum standardised uptake values (SUV_max_) of the radioligand. In US, quantified parameters are change in US backscatter before and after injection of microbubbles and represented as integrated area under the curve (IAUC) and rBF.

The biological features of a tumour, for example, vascularity, hypoxia, cell turnover determine its characteristics on functional imaging. The currently available imaging techniques available in the clinic for identifying tumour characteristics are MRI, PET, computerised tomography (CT) and US. Most of the functional techniques use an additional external contrast agent or radioactive tracer. Figure 1 summarises the commonly used imaging techniques and shows the quantitative readouts obtained.

### Imaging angiogenesis (blood flow and vascular permeability)

Perfusion imaging techniques exploit pathophysiological differences between ‘leaky’, disorganised, tumour neovessels and normal, well-organised vasculature. Serial images are acquired dynamically, through a volume of interest before, during and after administration of a contrast agent: gadolinium for MRI, iodinated contrast for CT and microbubbles for US. The acquired data are fitted to mathematical models to obtain quantitative parameters. The techniques are relatively simple to perform on standard clinical systems (1.5 Tesla for MRI, standard multidetector CT and most of the modern US machines) but require strict protocols, careful acquisition, accurate contrast agent dosing and injection rate, image timing, and image analysis for quantification.

The most common methods of perfusion imaging are dynamic contrast-enhanced (DCE) and dynamic susceptibility contrast (DSC) MRI, the latter being used almost exclusively in brain imaging. Magnetic resonance imaging sequences are designed to be sensitive to the presence of contrast medium in the extracellular space (EES) on DCE-MRI (T1 or relaxivity-based methods) or to the vascular phase of contrast medium delivery (T2 or susceptibility-based methods) on DSC-MRI. For DCE-MRI, the resulting signal intensity within the tumour reflects a composite of overall perfusion, vessel permeability or ‘leakiness’ and the volume of EES. A recent consensus meeting has recommended the following readouts: volume transfer constant of contrast between the blood plasma and the EES (*K*^trans^), size of the EES (*v*_e_) and IAUGC (area under the Gadolinium curve) for assessing antiangiogenic and vascular disrupting agents in clinical trials (Leach *et al*, 2005). *K*^trans^ is often used as a marker for the permeability of tumour vasculature. Related measures such as the rate constant (*k*_ep_), which describes the ratio of *K*^trans^/*v*_e,_ have also been used. For DSC-MRI, the degree of signal loss observed is dependent on the vascular concentration of the contrast agent and tumour perfusion; the most common used parameters are relative cerebral blood volume (BV), relative cerebral blood flow (BF) (Pope *et al*, 2011).

The data from DCE-CT imaging has a high spatial resolution and is potentially more quantitative than MRI as the change in CT image intensity is linearly related to the concentration of the contrast agent. The technique also benefits from FDA-approved software platforms embedded in standard CT reporting workstations. However, there is always a trade-off between the radiation dose and image quality and the contrast dose (potentially nephrotoxic) is relatively high, resulting in a limited use of CT for repeated scanning. The commonly used parameters include: BF, BV, MTT (mean transit time) and permeability (Goh *et al*, 2006).

Dynamic contrast enhanced-US has been used to measure haemodynamics in human tumours over the last decade. The contrast agent consists of phospholipid-based microbubbles with a mean diameter on the order of 0.5–10 *μ*m that encapsulate an inert gas, which when exposed to an US pulse, generate nonlinear resonances that allow enhanced representation of the vasculature. Microbubbles remain strictly intravascular following administration and therefore generated echoes relate to the flow within functional vessels. Most commonly, a 3-min acquisition is performed through a limited area within the tumour. The time-intensity curve in the region of interest depends on perfusion and is fitted to a kinetic model to determine volume and flow rate and semi-quantitative parameters such as peak intensity, time to peak intensity, MTT, area under the total enhancement curve (Lamuraglia *et al*, 2010), with most scanners offering data analysis software. However, DCE-US is still in its infancy and greatly operator dependent.

Amongst, positron-emitting tracers, H_2_^15^O-PET can be used to study tumour BF. Being a freely diffusible tracer, H_2_^15^O concentration shows a linear relation with signal intensity as measured with PET and the technique is thought to be able to absolutely quantify tumour perfusion (de Langen *et al*, 2008). A dynamic H_2_^15^O-PET scan acquisition takes 10 min and starts simultaneously with the bolus injection. The tracer has a short radioactive half-life of approximately 2 min, which allows repeated imaging after a short time or in combination with other tracers but requires an on-site cyclotron. The radiation dose is similar to that of ^18^F-fluorodeoxyglucose (^18^F-FDG)-PET.

### Imaging hypoxia

Hypoxia is a clinically important cancer hallmark, being associated with increased resistance to treatment and poor survival. ^18^F-labelled fluoromisonidazole (^18^F-FMISO) is the most extensively studied PET tracer for imaging tissue oxygenation. Its use in clinical practice has been hindered by its relatively poor signal to noise ratio because of slow specific accumulation as well as slow clearance from normoxic tissues (Mees *et al*, 2009). Next-generation PET tracers like ^18^F-labelled fluoroazomycin arabinoside have been developed and used successfully in head and neck cancer (Souvatzoglou *et al*, 2007).

Intrinsic susceptibility weighted (ISW)-MRI also known as BOLD (blood oxygenation level dependent)-MRI relies on the paramagnetic properties of deoxyhaemoglobin, to generate contrast because of the increased MR transverse relaxation rate (R2^*^). The technique is sensitive to pO2 within and in tissues adjacent to perfused vessels and can be easily quantified but does not measure pO2 directly. pH, carbon dioxide tension and haematocrit can affect R2^*^ readings and for correct interpretation of ISW-MRI the BV in the tissues of interest must be known (Padhani *et al*, 2007). More recently, it has been shown by analysing rapidly acquired DCE-MRI data with a specific kinetic model, it is also possible to obtain specific readouts of perfusion and permeability, which correlate with VEGF tumour expression and hypoxia (Donaldson *et al*, 2011).

### Imaging tumour cell proliferation, death and necrosis

Diffusion weighted-MRI exploits intrinsic contrast arising from the thermal displacement of water molecules over a given time. In biologic tissues, the movement of water molecules is hindered because their motion is limited by interactions with cell membranes and macromolecules. The quantified apparent diffusion coefficient (ADC), has been correlated with cellular density, presence of necrosis and in the therapy setting, tumour cell apoptosis and proliferation indices (Padhani *et al*, 2009). A low ADC reflects impeded diffusion and can be found in highly cellular tissues or fibrosis. If there are areas of significant necrosis this results in less restriction of motion and therefore a high ADC.

3′-Deoxy-3′-[^18^F]fluorothymidine (^18^F-FLT) PET had initial encouraging results as a potential biomarker for tumour proliferation. Currently, there is not enough data to support its use for reliable assessment of therapy response (Barwick *et al*, 2009).

### Imaging tumour metabolism

^18^F-fluorodeoxyglucose, a glucose analogue that allows mapping of tumour glucose use is widely used both in routine clinical practice and clinical trials. A decrease in the maximum standard glucose uptake value – SUV max, most commonly used quantitative parameter, has been associated with treatment response to numerous anticancer therapies (Shankar *et al*, 2006). More recently, it has been shown that Akt activation causes disruption to transcription of the glucose transporter GLUT1 and its translocation to the plasma membrane and promotes glucose utilisation independent of the effects on cell proliferation (Ma *et al*, 2009). ^18^F-FDG-PET has therefore been proposed as a pharmacodynamic biomarker for assessing efficacy of on-target inhibition of the PI3K/Akt pathway.

Pre and clinical hydrogen magnetic resonance spectroscopy (^1^H-MRS) studies have demonstrated that proliferating cells and many tumours display elevated choline signals. The method has been successfully used in characterisation and detection of brain and prostate cancer and there is some evidence towards its use as a response biomarker following chemotherapy in breast cancer (Baek *et al*, 2009). There is yet not enough evidence to support the role of MRS as a potential biomarker in phase I trials, although preclinical data are emerging (Beloueche-Babari *et al*, 2010), highlighting its potential.

## Evidence for use of functional imaging in pathway-specific targeted agents

### Measuring on-target effects on vasculature

Non-targeted microbubbles DCE-US after administration of a single dose (41 mg kg^–1^) of a combrestatin A4 derivative, AVE8062 in melanoma-bearing nude mice (which causes a rapid and selective shutdown of tumour neovasculature resulting in ischaemia and extensive haemorrhagic necrosis in xenografts) showed that intratumoural vessel depletion started 15-min post injection and was maximal at 6 h. This enabled planning of a clinical phase I assessment with DCE-US at 6- and 24-h post drug (Lavisse *et al*, 2008). Similar results were shown on clinical DCE-MRI, with the administration of CA4P causing a rapid reduction in *K*^trans^ and IAUGC at 4–6 h, which recovered by 24 h (Galbraith *et al*, 2003). A significant reduction in tumour BF and BV 30 min after treatment with CA4P (Anderson *et al*, 2003) has also been seen on H_2_^15^O-PET and ^15^O-CO PET. Notably, the effect on BV was still present at 24 h while the effect on BF was observed only in patients receiving larger doses of CA4P.

**Figure 2:**
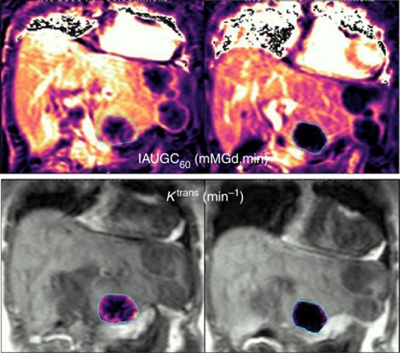
Dynamic contrast enhanced-MRI parametric maps before (left column) and 28 days after (right column) VEGF inhibitor therapy in a patient with metastatic colorectal carcinoma illustrating a down-stream effect with significant reduction in tumour vascularity (Courtesy to Dr C Messiou & M Orton, Royal Marsden Hospital).

The downstream effects of VEGFR inhibition on DCE-MRI have been documented in >40 phase I trials (Figure 2) with a significant reduction in *K*^trans^ and/or IAUGC being reported with multiple agents (Zweifel and Padhani, 2010). An interesting observation is that while the combination of VEGF and PDGF inhibitors has been associated with increased tumour vessel regression (Erber *et al*, 2004), the extent of associated change in DCE-MRI parameters with this combination of agents is variable or nonsignificant. This has been ascribed to the pro-permeability effects of PDGF inhibition by blocking pericytes recruitment to vessel walls and therefore reducing the vascular normalisation, and the associated reduction in *K*^trans^ (Erber *et al*, 2004).

A first phase I clinical trial using a humanised version of a mouse monoclonal anti-VEGF antibody HuMV833 labelled with ^124^I showed a marked heterogeneity in receptor expression both between and within patients with a three-fold variation in HuMV833 concentration within individual tumours indicating that response to these agents is likely to mirror this heterogeneity and therefore impact significantly on dose-response assessments (Jayson *et al*, 2002). Integrin labelled PET tracers and labelled microbubbles and paramagnetic nanoparticles coated with small peptidic *α*_v_*β*_3_ antagonists as MRI contrast agents have been described (Cai *et al*, 2005) but are still in experimental phase.

Correlations between reduction in vascularity on functional imaging such as DCE-MRI and DCE-US following antiangiogenic treatment and patient benefit have been reported in a small number of studies and warrant further exploration (Flaherty *et al*, 2008; Lassau *et al*, 2010; Jiang *et al*, 2012).

### Measuring off-target effects on vasculature

The early reduction of *K*^tran*s*^ on DCE-MRI in orthotopic mammary xenografts following treatment with a dual PI3K/mTOR inhibitor supports the biological observation that dysregulated angiogenesis and high tumour vascular permeability are in part PI3K dependent (Schnell *et al*, 2008). Although a decrease in the tumour perfusion by growth inhibition was reported on DCE-US following treatment with the mTOR inhibitor everolimus (5 mg kg^–1^ for 3 weeks) (Broillet *et al*, 2005), other data show that *K*^trans^ did not change in everolimus-treated tumour models when compared with measurable changes seen with the VEGF inhibitor vatalanib (Lane *et al*, 2009). However, the impact of mTOR inhibition with rapamycin analogues on the PI3K/Akt pathway is complex, and may actually involve increased Akt pathway activation (O'Reilly, 2006).

Inhibition of HIF-1*α* has dramatic effects on tumour vasculature: a dramatic reduction in tumour blood vessel permeability was seen on DCE-MRI in tumour-bearing mice within 2 h of treatment with PX-478 (Jordan *et al*, 2005).

### Measuring on-target effects on metabolism

On-target effects of EGFR inhibition have been explored with radionuclide labelled agents in PET, for example, ^124^I, ^11^C or ^18^F-gefitinib. ^18^F-FDG-PET response has been correlated with Akt inactivation and plasma membrane GLUT1 expression in a study with mTOR inhibitors (Ma *et al*, 2009).

### Measuring off-target effects on metabolism

The effect of antiangiogenic agents on ^18^F-FDG uptake has been explored with reduction in SUV_max_ being reported amongst others with sorafenib (Siemerink *et al*, 2008) and CA4P (Anderson *et al*, 2003).

In the clinic, downstream effects of EGFR inhibitors have been imaged with ^18^F-FDG-PET. Early changes in the SUV_max_ at 2 days after gefitinib treatment associated with progression-free survival (PFS) of 12 months were seen in a small study (Sunaga *et al*, 2008). ^18^F-FLT has also been found to predict response and patient outcome (positive and negative predictive values 92.9%) to EGFR inhibitors with a reduction in SUV_max_ of >10.4% at 7 days post gefitinib therapy predicting RECIST response on CT at 6 weeks (Sohn *et al*, 2008).

## Stratifying patients for targeted therapies using functional imaging

Functional imaging provides additional information on tumour characterisation, which could help in the selection of patients for targeted therapies. For example, in patients treated with gefitinib, a low baseline SUV of ^18^F-FDG has been associated with a higher response rate (53% *vs* 18%) and a prolonged PFS – median, 33.1 *vs* 8.6 weeks (Na *et al*, 2008). In renal cell carcinoma patients treated with sorafenib, a high baseline *K*^trans^ has been shown to have predictive value and prognostic value with elevated baseline *K*^trans^ being associated with response and longer PFS (Flaherty *et al*, 2008; Hahn *et al*, 2008). Identifying HER-2 overexpressing breast cancers for treatment with trastuzumab (Tolmachev, 2008) or imaging the oestrogen receptor status (Dehdashti *et al*, 2009) before treatment with aromatase inhibitors are other examples of using molecular imaging as a means of stratifying patients based on target recognition into likely responders and non-responders. Recent studies have demonstrated that in patients with ER+ breast cancer, a higher ^18^F-FES (16alpha-^18^F-fluoroestradiol-17beta) uptake at baseline is predictive of responsiveness to endocrine therapy (Dehdashti *et al*, 2009).

Use of a combination of tumour functional features also may prove to be helpful in patient stratification. For example, the presence of flow-metabolism mismatch has been measured using with a combination of H_2_^15^O-PET and ^18^F-FDG-PET. A low BF (and thus probably low tissue oxygenation) with a high tumour metabolism has been linked with aggressive cancer phenotypes in breast (Dunnwald *et al*, 2008) and pancreatic cancer (Komar *et al*, 2009); in the latter study SUV_max_/BF ratio was significantly higher in those living <12 months compared with those with survival >12 months, although neither parameter independently was significant between groups.

Identification of tumour hypoxia could facilitate the use of bioreductive drugs, which are metabolised to cytotoxic compounds only in hypoxic cells. Tirapazamine (TPZ) is the most advanced such drug in development and the relatively limited benefit in tumour control in >1100 patients was likely partially due to poor patient stratification with inclusion of patients with better-oxygenated tumours in the trials. A recent report comparing cisplatin/5-FU *vs* cisplatin/TPZ in which ^18^F-FMISO-PET was used to stratify the tumours into hypoxic and non-hypoxic subgroups showed that TPZ improved local control in hypoxic but not in non-hypoxic head and neck tumours (Rischin *et al*, 2006).

## Imaging to define dose and/or schedule

In the clinic, phase I trials usually incorporate a dose-escalation phase in which imaging does not formally have a role, under the assumption that the biggest imaging changes are likely to be detected at the highest dose, and the objective of this phase is to establish the MTD. However, with targeted agents functional imaging changes are likely to be apparent at lower doses than the MTD because of biological activity of the agent by direct target inhibition. A plateau level at which increasing dose is not followed by an imaging parameter change suggests that imaging may be helpful in choosing the optimal biological dose. Despite this, there are few cases in which imaging parameter changes have helped define the effective dose to take into phase II studies. Examples are *K*^trans^ change following treatment with sorafenib (Hahn *et al*, 2008) and brivanib (Jonker, 2011), H_2_^15^O-PET and C^15^O-PET information with CA4P treatment. In studies of CA4P, increasing effects on perfusion were noted to plateau at doses ⩾52 mg m^–2^ resulting in a recommended phase II dose of 52–66 mg m^–2^, below the MTD dose of 88 mg m^–2^ (Anderson *et al*, 2003).

Use of imaging to determine the optimal schedule of a targeted agent or for monitoring drug activity according to the class of therapeutic agent has received very little attention and data are limited. Jonker *et al* (2011) in a study of brivanib, a dual VEGFR and FGFR tyrosine kinase inhibitor, investigators evaluated DCE-MRI responses in several dose schedules in selected patients known to respond to anti-VEGF therapies and then selected the optimal schedule for a phase II trial. A similar approach has been utilised with sorafenib in renal cancer by Hahn *et al* (2008).

## Optimising and standardising functional imaging readouts

### Technique reproducibility

Better acquisition techniques for DCE-MRI, quality assurance and improved data modelling have meant that reproducibility figures have improved from over 30% to 26% for IAUGC and from >40% to around 30% for *K*^trans^ (Table 1).

Preliminary data for DW-MRI suggest that ADC measurements have a reproducibility of ∼15% in a multicentre setting (Koh *et al*, 2009) but it is most likely to range between 8 and 15%, depending on lesion location, acquisition plane and acquisition protocol.

For ^1^H-MRS reproducibility, data come mainly from brain studies: median coefficient of variance (CV) was 6.2% and 9.7% for serial measurements of NAA and choline, respectively (Maudsley *et al*, 2010). In the liver measurements of choline are challenged by large contaminant lipid peaks and patient motion.

Reported thresholds for ^18^F-FDG-PET for determining metabolic changes being approximately −34% to +52% for individual centres and −27% to +37% after centralised QA (Velasquez *et al*, 2009). For H_2_^15^O-PET, two small oncological studies, have reported wCV of 10–11% for breast and abdominal tumours (de Langen *et al*, 2008).

Other described techniques lack reproducibility data.

### Data analysis technique

**Figure 3:**
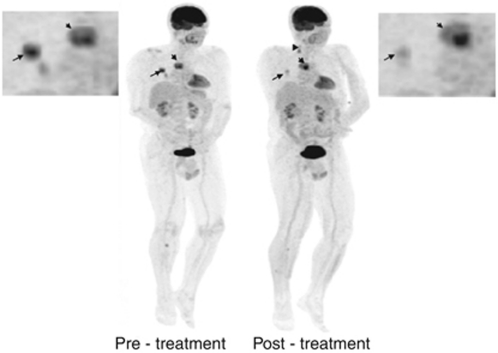
Differential response in ^18^F-FDG PET uptake after 1 cycle of targeted therapy with a bRaf and Mek inhibitor in a patient with metastatic melanoma. The mediastinal nodal mass has increased in size and demonstrates a heterogeneous increase in SUV (short arrow) while lung nodules are smaller in size and show significant reduction in SUV (long arrow). The tibial bony lesion seen on the pre-treatment scan is smaller, but a new tibial lesion is visible. The new cervical nodes (arrowhead) were felt to be inflammatory in aetiology.

Traditionally, a single target lesion was chosen for analysis based on its location, size and morphology (ideally >2.5 cm but <5 cm with <50% necrosis, in a location not greatly influenced by movement or artefacts). To account for differential response in phase I trials (Figure 3), identification of several lesions with different characteristics is preferred to include a spectrum of potential drug effects. This can be achieved with whole body scanning: whole-body PET is widely used and sliding table techniques with faster sequences in MRI have also enabled whole-body MRI in <60 min. Separate analysis of tumours subregions may also detect differential drug effects such as rim *vs* core differential effects in antiangiogenic therapy, which maybe missed on whole tumour analysis.

Summary parameters such as mean and median values oversimplify data and may mask critical information concerning tumour heterogeneity. Histogram, fractal and principal component analysis are viable alternatives. Parametric response maps (PRMs) are novel methods of image analysis using a voxel-by-voxel approach that can be applied to various functional imaging techniques such as perfusion or ADC maps. Two imaging volume data sets (e.g., baseline and early response) are co-registered and computationally analysed to yield PRM, which are generally colour coded based on the magnitude and direction of the parameter change. The changes can be displayed as histograms or scatter plots. In malignant brain tumours, PRM_ADC_ obtained 3 weeks and PRM_rBV_ and PRM_rBF_ at 1 and 3 weeks into conventional therapy was prognostic for later radiographic response, time to progression and overall survival (OS) whereas percentage change in mean parameters (ADC, relative BV and relative BF) was less significant (Galbán *et al*, 2009a). Similar findings have also been reported using PRM_ADC_ in head and neck tumours (Galbán *et al*, 2009b) and assessment of metastatic bony disease in prostate cancer (Reischauer *et al*, 2010). These methods of data analysis are still in early stages of development and evaluation.

### Multiparametric imaging

**Figure 4:**
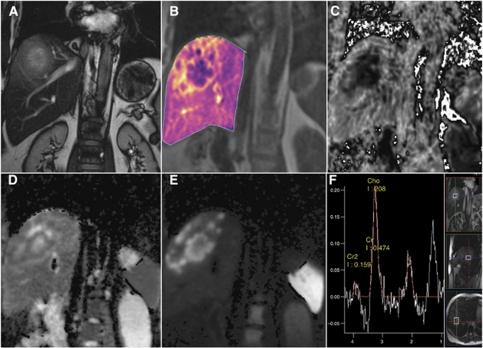
Multiparametric MRI protocol in the coronal plane in a patient with liver metastases from colon cancer showing a combination of anatomical information (T2-weighted, **A**) with functional imaging parametric maps, which quantify tumour vascularity (*K*^trans^, **B**), areas of hypoxia (R2^*^ maps, **C**) and tumour cellularity (ADC and DWI, **D** and **E,** respectively) together with single voxel ^1^H-MRS (**F**). The metastasis shows a vascular (**B**), hypercellular (**D** and **E**) rim with a hypoxic (**C**), necrotic (**D**) centre with increased proliferative activity as evidenced by choline signal in (**F**).

Targeted molecular approaches typically seek to inhibit specific pathways or molecules deemed to have an important role in tumour progression. Most of the preclinical data comes from relatively ‘homogenous’ cell lines and xenografts. In contrast, patients enrolled in phase I trials are heavily pre-treated and have heterogenous tumours that may behave differently from the original primary and vary between sites of disease. A multiparametric approach using a combination of imaging biomarkers is more informative than a single imaging biomarker; for example, a composite ‘vascular normalisation index’ integrating *K*^trans^, CBV and plasma collagen IV after one dosing of cediranib has been shown to correlate with PFS and OS in patients with recurrent glioblastoma (Sorensen *et al*, 2009). Multiparametric MRI data (DCE-MRI, DW-MRI, MRS and ISW-MRI) through a target volume can be acquired in 50–60 min (Figure 4).

### Defining criteria of response using functional imaging indices

Imaging criteria of response to date have relied on size evaluation as this has been shown to equate to improved clinical outcome. With functional imaging, new definitions of response need to be set and equated with PFS and OS. In the first instance, levels of change >95% confidence intervals of baseline variability (from reproducibility studies) for each technique need to be set in order to measure treatment-induced changes that specifically relate to drug activity. Although available studies are small and confounded by inter-patient heterogeneity, generally data show that patients whose tumours undergo at least a 50% reduction in DCE-MRI parameters attain stable disease or response by RECIST; also, DCE-MRI can differentiate tumour progression from treatment-induced changes in the brain with 95% sensitivity and 78% specificity (Narang *et al*, 2011).

Data on the expected magnitude of change in ADC indicative of response to standard therapy are becoming available for rectal, liver, lung and ovary tumours. It is likely that an increase in 20–25% after 1 week of treatment and at least 40% at later time points (3–4 weeks) of treatment will be indicative into tumour response (Sun *et al*, 2011). In bone, as with soft tissue tumours an increase in ADC occurs in responding lesions but because of the contribution of low ADC values from marrow fat in response, this requires more complex analysis (Messiou *et al*, 2011).

As with MRI, response assessment in ^18^F-FDG-PET requires a consistent methodology to allow quantitative assessments. EORTC guidelines published in 1999 suggested that medically relevant beneficial changes are often associated with a 30% or greater decline. PERCIST criteria that recommend SUV evaluation in the most active region of metabolically active tumours and include RECIST 1.1 in cases that do not have ^18^F-FDG avidity have been also been proposed (Wahl *et al*, 2009).

## Summary and conclusion

There is a clear need for robust functional imaging readouts to effectively assess novel cancer therapeutics and stratify patients to appropriate therapies. The tools to characterise a tumour phenotype on imaging based on vascularity, cellularity, metabolism and hypoxia exist but more specific targeted molecular profiling is still in its infancy. Development of dedicated tracers to identify receptor status will allow mapping of the whole tumour burden and its heterogeneity.

In the context of phase I trials, functional imaging should be performed at least in the expansion phase in order that it can inform and assist in the evaluation of target inhibition, patient selection and anti-tumour activity. Imaging methods should be relevant to the targeted pathway and specialist radiologist input is essential at the stage of trial design and set-up to ensure that the imaging protocol is designed accordingly.

We therefore recommend that perfusion imaging should be routinely incorporated into trials of agents targeting tumour vasculature as a biomarker of antiangiogenic activity and techniques such as ^18^F-FDG-PET should also be considered for early clinical trials of inhibitors of specific pathways, such as PI3K/AKT/MTOR. DW-MRI provides a biomarker for apoptosis and cell death and before enrolling a patient in a trial with a bioreductive agent, the hypoxic status of the tumour should be imaged with ^18^F-Miso. The evidence for other tracers such as ^18^FLT-PET does not warrant routine use. As receptor imaging techniques, (e.g., Her2 receptor status), mature they will prove invaluable for patient selection and treatment monitoring.

Currently, the use of functional imaging in large-scale multicentre clinical trials is prohibited by the limited technique reproducibility between centres and scanners, inadequate standardisation of data acquisition and analysis techniques and lack of necessary skills outside large centres. These obstacles are being addressed by programs such as the EU innovative medicine initiative in imaging, which should achieve standardisation of acquisition and analysis methodology for functional imaging biomarkers. There is an urgent need to strengthen the evidence base and increase familiarity with functional imaging techniques, through both single and multicentre studies.

## Figures and Tables

**Table 1:** Summary of the main functional imaging techniques used in phase I clinical trials in which a significant change in imaging parameters following treatment has been observed

**Technique**	**Coverage**	**Imaging principle**	**Biological feature explored**	**Imaging parameters (most used)**	**Reproducibility**	**Agent**	**Target**	**Time points at which a statistically significant change in imaging parameters has been observed**
DCE (dynamic contrast enhanced)-MRI	Single organ; 12–14 slices (6–7 cm)	Changes in T1 signal intensity after administration of low molecular weight contrast agent	Tissue perfusion and vascularity	IAUGC (Integrated area under Gadolinium curve)	wCV=12–16% * r*=26–30% (Morgan *et al*, 2006)	Axitinib (AG013736)	VEGFR1, 2; PDGFR-*β*	2 and 28 days (Liu *et al*, 2005)
						Cediranib (AZD2171)	VEGFR1,2,3; PDGFR-*α*, -*β* c-KIT	1 and 2 and 5 and 28 and 112 days (Batchelor *et al*, 2007; Drevs *et al*, 2007)
				*K*^trans^ (transfer constant)	wCV=19–29% *r*=30–36% (Morgan *et al*, 2006)	Sorafenib (BAY 43–9006)	Raf-1; wtBRAF; VEGFR2,3; PDGFR-*β*; FLT-3; KIT; FGFR-1	12 weeks (Flaherty *et al*, 2008) 2.7–10.9 weeks (median 6.1) (Hahn *et al*, 2008)
					wCV=24% (Galbraith *et al*, 2002a)	Intedanib (BIBF-1120)	VEGFR 1,2,3; FGFR 1, 2,3; PDGFR, Src, Lck, Lyn, FLT-3	3 and 30 days (Mross *et al*, 2010)
				*k*_ep_ (rate constant)	wCV=9% (Galbraith *et al*, 2002a)	Brivanib (BMS-582664)	VEGFR2, VEGFR3, FGFR1 FGFR2	2 and 8 and 26 days (Jonker *et al*, 2011)
						HuMV833	VEGF	48 h and 35 days (Jayson *et al*, 2002)
				*v*_e_ (extracellular space)		Vatalanib (PTK787/ZK-222584)	VEGFR 2	2 and 28 and 30days (Morgan *et al*, 2003)
						Sunitinib (SU11248)	VEGFR-1, -2; PDGFR*β*; KIT; FLT-4; c-FMS	14 days (Zhu *et al*, 2009)
						CA4P	Tubulin polymerization;	4–6 h and 24 h (Dowlati *et al*, 2002)
						Vadimezan (DMXAA)	Established tumour blood vessels	24 h (Galbraith *et al*, 2002b)
						ZD6126	Colchicin analogue (tubulin binding)	24 h (Evelhoch *et al*, 2004)
DCE-CT	Single organ 4–8 slices (2.5–5 mm)	Changes in CT density (HU) following administration of iodinated contrast agent	Tissue perfusion and vascularity	rBV (relative blood volume)	wCV 14–15% * r*=38% (Goh *et al*, 2006)	Bevacizumab	Humanized anti-VEGF monoclonal antibody	10–12 days (Jiang *et al*, 2012)
						Sorafenib (BAY 43-9006)	Raf-1; wtBRAF; VEGFR-2, -3; PDGFR-*β*; FLT-3; KIT; FGFR-1	6 weeks (Fournier *et al*, 2010)
				rBF (relative blood flow)	wCV=23% * r*=65% (Goh *et al*, 2006)	Sunitinib (SU11248)	VEGFR1,2,3; PDGFR-*α*, -*β*; KIT; FLT-3; CSFR-1; RET	6 weeks (Fournier *et al*, 2010)
				MTT (mean transit time )	wCV=35% * r*=97% (Goh *et al*, 2006)			
DCE-US	Single lesion single slice	Enhanced representation of the vasculature following administration of microbubbles	Tissue perfusion and vascularity	AUC (area under curve)	No data available in clinical studies	Bevacizumab	Humanized anti-VEGF monoclonal antibody	3 and 7 and 14 and 60 days (Lassau *et al*, 2011)
				BF (blood flow)		Sorafenib (BAY 43-9006)	Raf 1; wtBRAF; VEGFR-2, -3; PDGFR-*β*; FLT-3; KIT; FGFR-1	3 and 6 weeks (Lamuraglia *et al*, 2006)
				PI (peak intensity)		Sunitinib (SU11248)	VEGFR-1, -2,-3; PDGFR-*α*, *β*; KIT; FLT-3; CSFR-1; R	15 days (Lassau *et al*, 2010)
				TPI (time to PI)				
Diffusion Weighted Magnetic Resonance Imaging (DW-MRI)	Whole organ coverage routinely; whole body diffusion available	Measures water tissue diffusibility by applying two, balanced, opposing magnitude, gradient pulses to a conventional T2w, spin-echo MRI sequence	Indirect assessment of tissue cellularity and presence of necrosis	ADC) (apparent diffusion coefficient)	*r*%=13.3 (Koh *et al*, 2009) two centre trial	CA4P Cediranib (AZD 2171)	Tubulin polymerization VEGFR1,2,3; PDGFR-*α*, -*β* c-KIT	3 h after 2nd dose (Koh *et al*, 2009) 1 and 28 and 56 days (Batchelor *et al*, 2007)
								
^18^F-FDG (glucose analogue) PET	Whole body	^18^F-FDG enters the cell via glucose transporters, phosphorylated by hexokinase and then trapped within cells.	Glucose utilization in tumour cells.	SUV max (standardized unit value)	wCV=10.7–15.9% * r*=−(34–27)% to +(37–52)% multicenter data	Sorafenib (BAY 43-9006)	Raf-1; wtBRAF; VEGFR-2, -3; PDGFR-*β*; FLT-3; KIT; FGFR-1	3 weeks (Siemerink *et al*, 2008)
					*r*=6–10–42%	Sunitinib (SU11248)	VEGFR-1, -2,-3; PDGFR-*α*, -*β*; KIT; FLT-3; CSFR-1; RET	10–14 day (Van den Abbeele *et al*, 2008)
					Single centre data acquisition (Nahmias and Wahl, 2008; Velasquez *et al*, 2009)	rh-Endo recombinant human endostatin	Proliferation and migration of capillary endothelial cells	28 and 56 days (Herbst *et al*, 2002)
				SUV mean	1–7% (Nahmias and Wahl, 2008)	Gefitinib	EGFR	2 days and 4 weeks (Sunaga *et al*, 2008)
^18^F-FLT (flurothymidine) PET	Whole body	Enter cells via nucleoside transporter proteins, phosphorylated by thymidine kinase, trapped intracellularly, but not incorporated into DNA	Tissue proliferation rate	SUV 41%	ICC=0.98 (95% CI 0.95–0.99) * r*=15%	Sunitinib	VEGFR-1, -2,-3; PDGFR-*α*, -*β*; KIT; FLT-3; CSFR-1; RET	4 weeks (Liu *et al*, 2011)
				SUV max	ICC=0.93 (95% CI 0.85–0.97) * r*=20–25% (de Langen *et al*, 2009)			
H2^15^O- (labeled H_2_O) PET	Whole body	Inhaled C^15^O_2_ or intravenous H_2_^15^O. reach an equilibrium in which the diffusion rate into the tissue from the arterial blood is balanced by the diffusion rate out of the tissue into venous blood and the rate of radioactive decay of the ^15^O:	Tissue blood flow and oxygen utilization	Tumour perfusion	*r*=15.8–40% (depending on VOI selection method) (van der Veldt *et al*, 2010)	rh-Endo recombinant human endostatin	Proliferation and migration of capillary endothelial cells	28 and 56 days (Herbst *et al*, 2002)
						CA4P	Tubulin polymerization	24 h (Anderson *et al*, 2003)
				Regional flow	wCV 11% (van der Veldt *et al*, 2010)			
				Volume of distribution (VT or V_d_)	*r*=32% (van der Veldt *et al*, 2010)			
					*r*=36–47% (de Langen *et al*, 2008)			

Abbreviations: ADC=apparent diffusion coefficient; AUC=area under curve; BF=blood flow; CI=confidence interval; CT=computerised tomography; CV=coefficient of variance; DCE=dynamic contrast enhanced; DW=diffusion weighted; 18F-FDG=18F-fluorodeoxyglucose; 18F-FLT=18F-fluorothymidine; HU=hounsfield unit; ICC=interclass correlations; IAUGC=integrated area under gadolinium curve; KIT=mast/stem cell growth factor receptor; MRI=magnetic resonance imaging; MTT=mean transit time; PET=positron emission tomography; PDGFR=platelet derived growth factor receptor; PI=peak intensity; r=reproducibility coefficient; RAF1, BRAF=members of the raf kinases family (raf - rapidly accelerated fibrosarcoma); rBF=relative blood flow; rBV=relative blood volume; SUV=standardised unit value; TPI=time to PI; VEGFR=vascular endothelial growth factor receptor; US=ultrasound; VOI=volume of interest; wtBRAF=wild type BRAF.
